# Supplementary material for: The timing of differentiation and potency of CD8 effector function is set by RNA binding proteins
Source: Nat Commun. 2022 Apr 27;13:2274. doi: 10.1038/s41467-022-29979-x (PMC9046422; doi:10.1038/s41467-022-29979-x)
Supplement: Supplementary file 3 — Description of Additional Supplementary Files [file 41467_2022_29979_MOESM3_ESM.pdf]

Description of Additional Supplementary Files

Supplementary Data 1

List of RBP target genes grouped into clusters I-V, which are dynamically regulated upon naïve $CD8^+$ T cell activation.

Significantly differentially expressed genes at 6 and 18 hours after T cell activation were included using a log2FC>1.3 or <-1.3 cut off and an adjusted p value <= 0.05 (analysed by DESeq2; Wald test followed by Benjamini-Hochberg correction for adjusted p-values). Genes are grouped according to their molecular and physiological function within each cluster. Cluster I contains the list of target genes which are induced 6 hours after activation. Cluster II contains target genes which are induced 6 and 18h after activation. Cluster III contains target genes which are only induced at 18 after activation. Cluster IV and V contain genes which show reduced expression compared to unstimulated cells 6h or 18h after activation respectively.
