# Supplementary material for: The timing of differentiation and potency of CD8 effector function is set by RNA binding proteins
Source: Nat Commun. 2022 Apr 27;13:2274. doi: 10.1038/s41467-022-29979-x (PMC9046422; doi:10.1038/s41467-022-29979-x)
Supplement: Supplementary file 5 — Reporting Summary [file 41467_2022_29979_MOESM5_ESM.pdf]

nature portfolio

Corresponding author(s): Martin Turner

Last updated by author(s): 2022/02/21

Reporting Summary

Nature Portfolio wishes to improve the reproducibility of the work that we publish. This form provides structure for consistency and transparency in reporting. For further information on Nature Portfolio policies, see our Editorial Policies and the Editorial Policy Checklist.

Statistics

For all statistical analyses, confirm that the following items are present in the figure legend, table legend, main text, or Methods section.

n/a	Confirmed	
☐	☒	The exact sample size (n) for each experimental group/condition, given as a discrete number and unit of measurement
☐	☒	A statement on whether measurements were taken from distinct samples or whether the same sample was measured repeatedly
☐	☒	The statistical test(s) used AND whether they are one- or two-sided<br>*Only common tests should be described solely by name; describe more complex techniques in the Methods section.*
☒	☐	A description of all covariates tested
☒	☐	A description of any assumptions or corrections, such as tests of normality and adjustment for multiple comparisons
☐	☒	A full description of the statistical parameters including central tendency (e.g. means) or other basic estimates (e.g. regression coefficient) AND variation (e.g. standard deviation) or associated estimates of uncertainty (e.g. confidence intervals)
☒	☐	For null hypothesis testing, the test statistic (e.g. F, t, r) with confidence intervals, effect sizes, degrees of freedom and P value noted<br>*Give P values as exact values whenever suitable.*
☒	☐	For Bayesian analysis, information on the choice of priors and Markov chain Monte Carlo settings
☒	☐	For hierarchical and complex designs, identification of the appropriate level for tests and full reporting of outcomes
☒	☐	Estimates of effect sizes (e.g. Cohen's d, Pearson's r), indicating how they were calculated

Our web collection on statistics for biologists contains articles on many of the points above.

Software and code

Policy information about availability of computer code

Data collection	Publicly available datasets were downloaded from SRA using URLs obtained with SRA explorer (https://ewels.gitcf hub.io/sra-explorer/), inputting SRP accessions from the GEO records. iCLIP data sequenced in-house was processed using the standard Illumina pipeline for basecalling and demultiplexing.

Data analysis	RNA-seq data were trimmed using Trim Galore v 0.4.5, and mapped to the GRCm38 mouse genome using Hisat2 v2.1.0. Read counts over mRNA features from the GRCm38 v 90 annotation release were quantified with Seqmonk v1.41.0, using the RNA-seq quantitation pipeline, merging isoforms. DESeq2 analysis was performed using R v 3.5.3 and DESeq2 v1.20.0.<br>Raw fastq files for CLIP data were uploaded to the iMaps platform (currently hosted by Genialis: https://imaps.genialis.com/iclip), and analysis performed using the iCount pipeline (v2.0.1dev); documentation for this software and instructions for local installation are provided here: https://icount.readthedocs.io/en/latest/. Versions of the external software called by the pipeline were: Cutadapt v2.4, STAR v2.7.0f.<br>Downstream analysis of CLIP data was performed using R v4.0.4.<br>Scripts used to annotate the CLIP data, assigning genes and features to each crosslink site, and combining replicates to find sites significant in multiple replicates, together with instructions and sample data allowing these to be tested, have been added to a github repository available here:<br>https://github.com/LouiseMatheson/Process_CLIP_data<br>Underlying code, instructions and test data for a shiny app for visualisation of the CLIP data, together with sample code showing how the data is preprocessed, are available in a github repository here:<br>https://github.com/LouiseMatheson/iCLIP_visualisation_shiny<br>The sample data provided here comprise the ZFP36L1 replicate CLIP datasets, over a region encompassing the Tnf gene, and thus allow reproduction of part of Supplementary Figure 4C from the manuscript.<br>Flow cytometry data was analysed using FlowJo version 10.6 and Graph Pad Prism 8.1.2..

For manuscripts utilizing custom algorithms or software that are central to the research but not yet described in published literature, software must be made available to editors and reviewers. We strongly encourage code deposition in a community repository (e.g. GitHub). See the Nature Portfolio guidelines for submitting code & software for further information.

Data

Policy information about availability of data

All manuscripts must include a data availability statement. This statement should provide the following information, where applicable:

- Accession codes, unique identifiers, or web links for publicly available datasets
- A description of any restrictions on data availability
- For clinical datasets or third party data, please ensure that the statement adheres to our policy

All data generated and analysed during this study are included in this article and its supplementary information, or have been made available in public repositories as follows:
Sequencing data from ZFP36L1 iCLIP experiments performed in this manuscript is publicly available on GEO: GSE176313.
Previously published datasets are available on GEO under the accessions GSE77857 (RNA-seq) and GSE96074 (HITS-CLIP for ZFP36).

Field-specific reporting

Please select the one below that is the best fit for your research. If you are not sure, read the appropriate sections before making your selection.

☒ Life sciences ☐ Behavioural & social sciences ☐ Ecological, evolutionary & environmental sciences

For a reference copy of the document with all sections, see nature.com/documents/nr-reporting-summary-flat.pdf

Life sciences study design

All studies must disclose on these points even when the disclosure is negative.

Sample size	Sample sizes have not been determined in advance due to the novelty if this research and no prior knowledge of the effect size of the genotype.
Data exclusions	Data was not excluded
Replication	Experiments have been repeated atleast twice and data has shown reproducibility except for Cas9 mediated KO experimen which has been done once with 4 biological replicates
Randomization	Allocation of mice into experimental groups was random within the margins of experimental design (genotype of mice, age of mice). Mice within the same age group or genotype were randomly allocated to experimental groups. Samples were analyzed and acquired in a randomized fashion.
Blinding	In vivo transfer experiments were performed by animal experimental facility staff in a blinded fashion. For all other experiments the investigators were not blinded to group allocation or sample collection since the experiments were all designed and performed by the same investigating person.

Reporting for specific materials, systems and methods

We require information from authors about some types of materials, experimental systems and methods used in many studies. Here, indicate whether each material, system or method listed is relevant to your study. If you are not sure if a list item applies to your research, read the appropriate section before selecting a response.

Materials & experimental systems

n/a	Involved in the study	
☐	☒	Antibodies
☐	☒	Eukaryotic cell lines
☒	☐	Palaeontology and archaeology
☐	☒	Animals and other organisms
☒	☐	Human research participants
☒	☐	Clinical data
☒	☐	Dual use research of concern

Methods

n/a	Involved in the study	
☒	☐	ChIP-seq
☐	☒	Flow cytometry
☒	☐	MRI-based neuroimaging

Antibodies

Antibodies used

Specificity clone fluorochorme supplier cat no concentration
CD4 GK1.5,RM4-5 Fitc/biotin/BV605 eBioscience/BioLegend 13-0041-85;100510;100548 (1:400)
Cre Recombinase DL7L7L PE Cell Signalling 59238 (1:50)
CD8 53-6.7 AF700/APC BD Pharmingen/eBioscience 557959:17-0081-82 (1:400)
CD45.1 A20 eF450/BUV395 eBioscience/BD Pharmingen 48-0453-82;565212 (1:100)
CD45.2 104 Fitc/BV785 BioLegend 109806;109839 (1:100)
CD45 30-F11 Fitc eBioscience 11-0451-81 (1:100)
KLRG1 2F1 BV605/BV421/BUV737 BioLegend/BD Pharmingen 138419;562897/BDB741812 (1:400)
CD127 A7R34 PE-Cy7/APC BioLegend 135014/135012 (1:100)
CD44 IM7 Bio/BV510/ PercpCy5.5 BD Pharmingen/BioLegend/eBioscience 553132;103043;103032 (1:400)
CD25 PC61 BV650/PECy7 BioLegend/eBioscience 102038;25-0251-82 (1:400)
CD69 H1.2F3 BV786 BD Pharmingen 564683 (1:400)
MHCII M5/11415.2 Fitc eBioscience 11-5321-82 (1:400)
TCRβ H57-597 PE-Cy7/PercpCy5.5 BioLegend/eBioscience 109222;45-5961-80 (1:200)
CD11c cN418 Fitc/biotin BioLegend/Tonbo 117306;30-0114-U500 (1:400)
CD11b M1/70 Fitc/biotin eBioscience 11-0112-41 ;13-0112-82 (1:400)
F4/80 BM8 Fitc/biotin eBioscience/BioLegend 11-4801-82; 123106 (1:400)
NK1.1 PK136 Fitc/biotin eBioscience/BD Pharmingen 11-5941-82;553163 (1:400)
CD19 6D5/1D3 Fitc/biotin BioLegend/BD Pharmingen 115506;553784 (1:400)
gdTCR GL3 biotin eBioscience 13-5711 (1:400)
CD105 MJ7/18 biotin BioLegend 120404 (1:400)
GR1 RB6-8C5 biotin eBioscience 13-5931-82 (1:400)
B220 RA3-6B3 biotin eBioscience 13-0452-82 (1:400)
Ter119 TER119 biotin eBioscience 13-5921-82 (1:400)
Thy1.1 OX7 PE BioLegend 202524 (1:200)
TNF MP6-XT22 AF488/BV650 BioLegend 506313;506333 (1:200)
IFN-γ XMG1.2 PE/AF488/AF647 BioLegend/eBioscience/BD Pharmingen 505808;53-7311-82;557735 (1:400)
IL2 JES6-5H4 PE BioLegend 503808 (1:100)
GranzymeB GB11 AF647/AF700 BioLegend/BD Pharmingen 515406/560213 (1:200)
c-REL 1RELAH5 PE eBioscience 12-6111-80 (1:200)
NFKB2 EPR18756 abcam ab191594 (1:200)
IRF8 V3GYWCH APC eBioscience 17-9852-80 (1:100)
NOTCH1-IC mN1A PE BioLegend 629106 (1:50)
EOMES Dan11mag AF488 eBioscience 11-4877-42 (1:400)
ZFP36 3D10 Origene TA500621 (1:500)
ZFP36L1 BRF1/2 Cell signaling #2119 (1:1200)
CD16/32 2.4G2 BioXcell BE0008 (1:2000, 0.5ug/ml)
polyclonal goat anti Rabbit (H+L) AF488 (A11034, Invitrogen;1:1000)

Validation

We have validated the antibodies for ZFP336, ZFP36L1, TNF, IFNg and IL2 using CrispR mediated deletion in T cells. For all other primary antibodies we have relied on validation provided by the supplier.

Eukaryotic cell lines

Policy information about cell lines

Cell line source(s)	platE cells (T.Kitamura, 2006), EL4 mouse T-cell precursor cell line (ATCC TIB-39)
Authentication	EL4 mouse cell line was validated by flow cytometry. PlatE cells were not authenticated
Mycoplasma contamination	Cell lines have been tested for mycoplasma contamination and found negative

Commonly misidentified lines (See ICLAC register) | no commonly misidentified cell lines were used in this study

Animals and other organisms

Policy information about studies involving animals; ARRIVE guidelines recommended for reporting animal research

Laboratory animals	mice (mus musculus). B6.SJL-Ptprca Pepcb, C57BL/6 mouse strains were used. Mice were between 8 and 12 weeks of age and male as well as female mice were used in the studies described here. The original publications describing the generation of the genetically altered alleles used in this study are given in the manuscript methods section.
Wild animals	no wild animals were used
Field-collected samples	no field samples were collected
Ethics oversight	Babraham Institute Animal Welfare and Ethical Review Body and UK Home Office licensing.

Note that full information on the approval of the study protocol must also be provided in the manuscript.

Flow Cytometry

Plots

Confirm that:

- [x] The axis labels state the marker and fluorochrome used (e.g. CD4-FITC).
- [x] The axis scales are clearly visible. Include numbers along axes only for bottom left plot of group (a 'group' is an analysis of identical markers).
- [x] All plots are contour plots with outliers or pseudocolor plots.
- [x] A numerical value for number of cells or percentage (with statistics) is provided.

Methodology

Sample preparation	Single cell suspensions were isolated from tissue of interest (spleen, lung, blood) using Buffer containing 1%FCS and 2mM EDTA. Samples were mechanically homogeneised and single cell suspensions were obtained and filtered by 40um filter. Work was performed with cold buffers and cells maintained on ice.
Instrument	BD Fortessa Flow Cytometer
Software	Flow Jo 10.6
Cell population abundance	Frequency of sorted naive CD8 T cells was between 10-20% of total lymphocyte population. Cells were either isolated by FACS sorting or using enrichment beads. Cell purity was confirmed by Flow cytometry.
Gating strategy	For all data sets cells were analyzed using forward and side scatter properties to select for lymphocytes and exclude doublets. Dead cells were excluded from analysis using a fixable viability dye.

- [x] Tick this box to confirm that a figure exemplifying the gating strategy is provided in the Supplementary Information.
